# Hepatotoxicity Profile of Bortezomib-Containing Regimens for First-Line Multiple Myeloma Treatment

**DOI:** 10.3390/ph19081205

**Published:** 2026-08-01

**Authors:** Marko Lucijanic, Jerko Orec, Ena Soric, Anica Sabljic, Zeljko Jonjic, Mario Pirsic, Ozren Jaksic

**Affiliations:** 1 Hematology Department, University Hospital Dubrava, Avenija Gojka Suska 6, 10000 Zagreb, Croatia; 2 School of Medicine, University of Zagreb, Salata 3, 10000 Zagreb, Croatia; 3 Scientific Research and Translational Medicine Department, University Hospital Dubrava, Avenija Gojka Suska 6, 10000 Zagreb, Croatia

**Keywords:** multiple myeloma, bortezomib, hepatotoxicity, drug-induced liver injury, proteasome inhibitor, overall survival, drug safety

## Abstract

**Background/Objectives:** Bortezomib, a proteasome inhibitor, is one of the key agents in the first-line treatment of multiple myeloma (MM). Although its hepatotoxic potential has been described in case reports and registration trials, systematic real-life studies quantifying the incidence, dynamics and prognostic significance of liver injury during bortezomib-based therapy are lacking. We aimed to characterize the hepatotoxicity profile of first-line bortezomib-based regimens. **Methods:** We retrospectively analyzed 115 consecutively treated MM patients at University Hospital Dubrava (2016–2023) who received first-line bortezomib-based regimens (VCD, VD, VMP, VRD, VTD). Liver function parameters (bilirubin, albumin, AST, ALT, GGT, ALP) were recorded at baseline and after 1 and 3 months, and abnormalities were graded according to CTCAE v5.0. Predictors of liver injury and its association with IMWG response and overall survival (OS) were assessed. **Results:** Elevation of any liver transaminase was observed in 33% of patients at baseline, 40.2% at 1 month and 24% at 3 months. De novo worsening developed in 23.4% of patients, predominantly (88%) within the first month, with a dominantly cholestatic pattern (96%). Liver injury was not associated with the antimyeloma regimen, the class of supportive therapy, or most comorbidities (besides second malignancy for 3-month derangement), whereas higher tumor burden and baseline liver injury were identified among the predictors. Transaminase abnormalities were not associated with the likelihood of achieving a response, but liver injury at 3 months independently predicted shorter OS (HR 2.83; *p* = 0.011) after adjustment for age, sex and ISS stage. **Conclusions:** Liver injury in bortezomib-treated MM patients is frequent and predominantly associated with the biology of the underlying disease rather than necessarily with the antimyeloma therapy itself; abnormalities persisting at 3 months deserve attention as an independent adverse prognostic marker.

## 1. Introduction

Multiple myeloma (MM) is a malignant clonal proliferation of plasma cells in the bone marrow, accounting for approximately 10% of all hematological malignancies and predominantly affecting older adults, with a median age at diagnosis of around 70 years [1]. The neoplastic plasma cells secrete a monoclonal immunoglobulin or its light-chain fragments and drive the characteristic end-organ damage captured by the CRAB criteria (hypercalcemia, renal insufficiency, anemia and osteolytic bone lesions), which, together with the SLiM biomarkers, define the threshold for initiating therapy [2]. Over the past two decades the prognosis of MM has improved substantially with the introduction of proteasome inhibitors, immunomodulatory drugs (IMiDs) and, more recently, anti-CD38 monoclonal antibodies [1,3].

Contemporary management is built upon targeted agents with potent biological activity against malignant plasma cells. Bortezomib and the IMiDs (thalidomide, lenalidomide, pomalidomide) form the backbone of first-line treatment, and daratumumab has progressively become an integral component of therapeutic protocols [3]. In Croatia, reimbursement of daratumumab through the national health insurance fund (Croatian Health Insurance Fund) was covered for second and later lines of therapy from 2019, while coverage for first-line treatment of patients who are not candidates for autologous hematopoietic stem cell transplantation (ASCT) was introduced only in 2024. Throughout the period preceding this change, bortezomib-based induction regimens represented the standard of care for first-line treatment.

Patients with MM frequently receive multiple concomitant supportive medications, including bisphosphonates and other bone-modifying agents, cardiovascular drugs, antimicrobials, and analgesics, each of which carries a variable intrinsic hepatotoxic potential. Given the advanced age, comorbidity burden and need for supportive therapy in this population, patients are exposed to a large number of different drugs and a high degree of polypharmacy. The relationship between treatment-related oxidative stress in myeloma and hepatic injury pathways has been highlighted in experimental and disease-model studies [4,5].

Hepatotoxicity has been recognized as an adverse event associated with bortezomib, manifesting mainly as laboratory elevations of serum transaminases, although its precise incidence in pivotal clinical trials has not been consistently reported [6,7,8]. In addition, its precise incidence in everyday clinical practice, the temporal dynamics of these changes and the predictors of liver injury remain poorly characterized, as the available evidence is largely limited to individual case reports [9,10] and pharmacovigilance summaries [11], while real-life cohort studies are practically non-existent. IMiDs, particularly lenalidomide, have been associated with hepatotoxicity in approximately 15% of patients in clinical trials, with serious events including hepatic failure reported in around 2% of patients with multiple myeloma [12,13,14], whereas cyclophosphamide, melphalan, dexamethasone and prednisone are usually associated with a mild hepatotoxic profile at the doses used in antimyeloma protocols [15,16,17].

Among the supportive drugs to which these patients are exposed, several agents with hepatotoxic potential may be present, for example trimethoprim-sulfamethoxazole, azole antifungals and allopurinol, which are documented causes of clinically apparent drug-induced liver injury [18]. The reference resource for verifying and classifying the potential hepatotoxicity of drugs is the LiverTox database [19], and the severity of hepatic adverse events can be graded according to the Common Terminology Criteria for Adverse Events (CTCAE) version 5.0 of the U.S. National Cancer Institute [20].

We hypothesized that the occurrence of hepatotoxicity in patients with MM differs depending on the type of administered therapy. The aim of this study was to determine the incidence of hepatotoxicity in MM patients treated with first-line bortezomib-based regimens, to characterize the temporal dynamics of liver function abnormalities, to investigate their relationship with the antimyeloma and supportive therapies to which patients are exposed and with individual comorbidities, and to assess the clinical significance of liver injury for treatment response and overall survival.

## 2. Results

### 2.1. Patient Characteristics and Profile of Antimyeloma and Supportive Therapy

**Figure 1:**
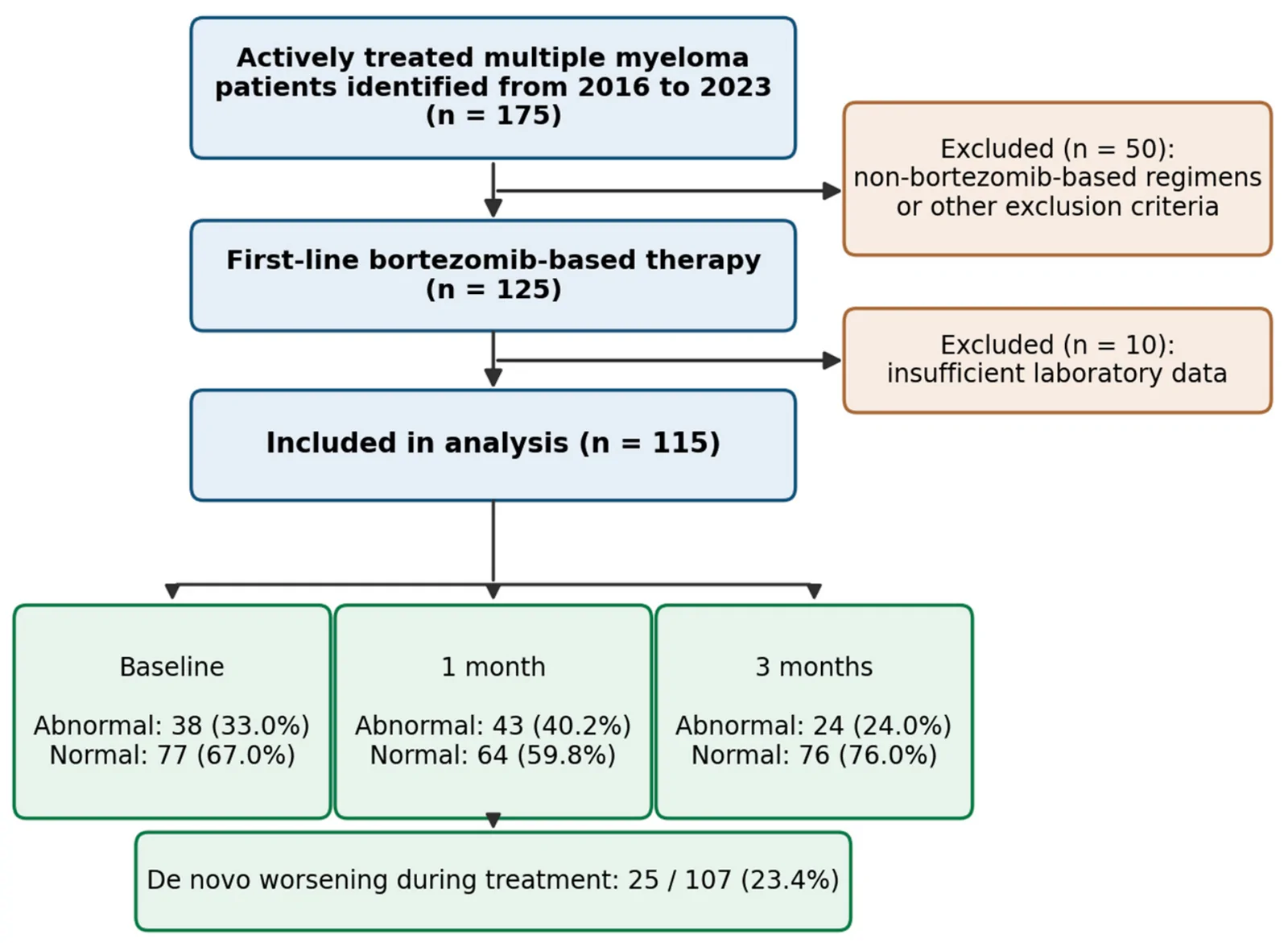
Flow chart of the study.

We analyzed a total of 115 patients with MM initiating first-line treatment with bortezomib-based regimens. The clinical and laboratory characteristics of the patients are presented in Table 1, and their comorbidities in Supplementary Table S1. The flow chart of patient selection and the distribution of liver function status at each time point are summarized in Figure 1.

There were 63 (54.8%) men and 52 (45.2%) women, with a median age of 68 years (interquartile range, IQR 60–74). According to the heavy-chain type, 74/113 (65.5%) patients had IgG, 19/113 (16.8%) IgA and 17/113 (15.0%) light-chain myeloma. According to the light-chain type, kappa was present in 73/111 (65.8%) and lambda in 37/111 (33.3%) patients. According to the International Staging System (ISS), 20/95 (21.1%) patients were in stage 1, 24/95 (25.3%) in stage 2 and 51/95 (53.7%) in stage 3.

**Figure 2:**
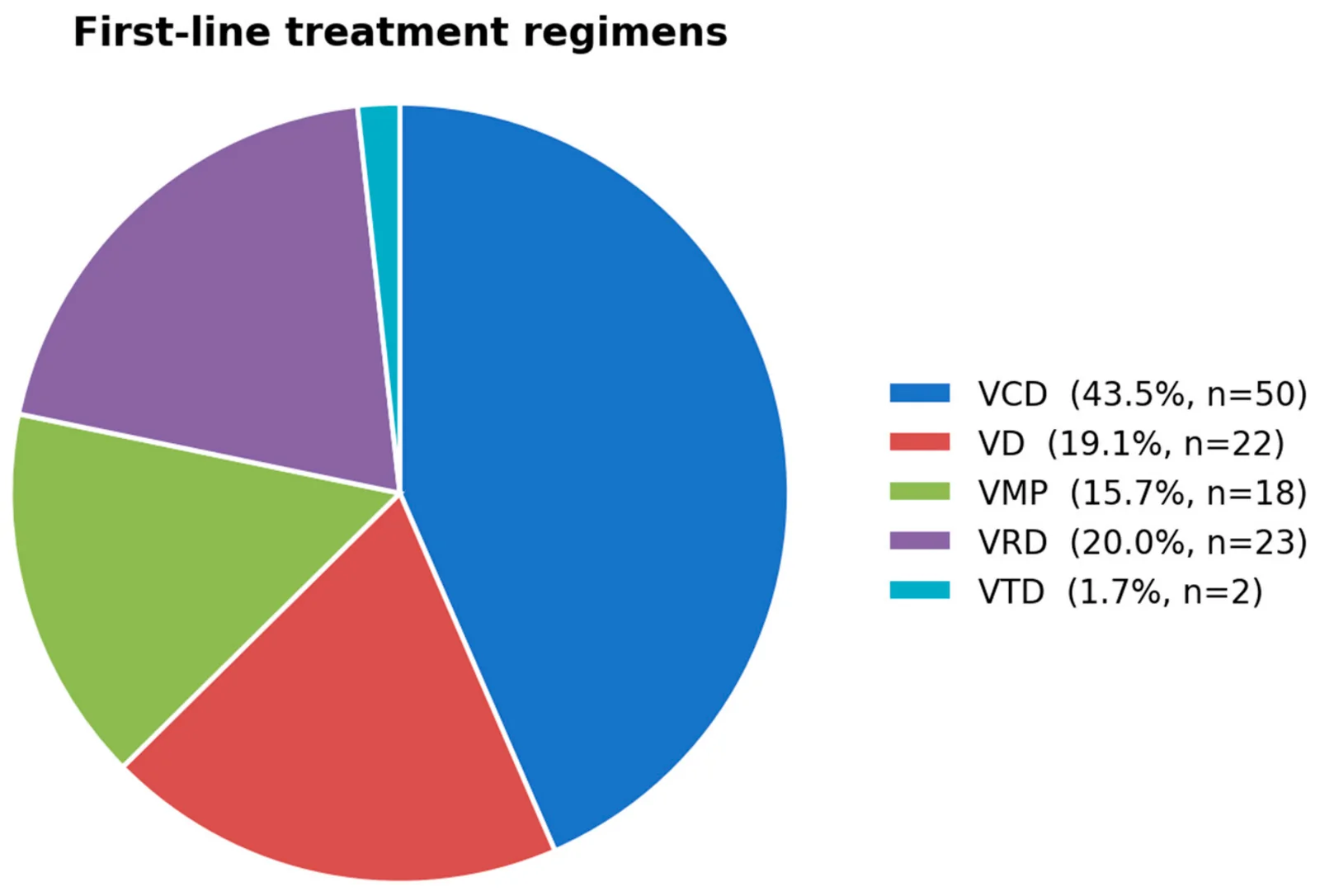
Distribution of patients according to the regimens used in first-line treatment. VCD: bortezomib, cyclophosphamide, dexamethasone; VD: bortezomib, dexamethasone; VMP: bortezomib, melphalan, prednisone; VRD: bortezomib, lenalidomide, dexamethasone; VTD: bortezomib, thalidomide, dexamethasone.

A total of 25/115 (21.7%) patients were treated with IMiD-containing regimens: 23/115 (20.0%) received VRD and 2/115 (1.7%) received VTD. The remaining 90/115 (78.3%) received non-IMiD regimens: 50/115 (43.5%) received VCD, 22/115 (19.1%) VD and 18/115 (15.7%) VMP. The distribution of patients according to first-line regimen is shown in Figure 2. ASCT was performed in 38/111 (34.2%) patients, comprising a single transplantation in 26/111 (23.4%) and tandem transplantation in 12/111 (10.8%).

After completion of first-line treatment, 21.2% of patients achieved complete response (CR), 34.8% very good partial response (VGPR), 13.6% partial response (PR), 7.6% stable disease (SD) and 22.7% progressive disease (PD). The median follow-up was 42 months, and a total of 40% patients died. The median overall survival was 43 months.

The profile of supportive therapy administered during the first three months is presented in Supplementary Table S2. According to the hepatotoxicity risk classification, 78/113 (69.0%) patients received supportive drugs of high risk, 31/113 (27.4%) of moderate risk and 4/113 (3.5%) of low risk for the development of hepatotoxicity.

### 2.2. Liver Function Profile at Baseline and After 1 and 3 Months of Antimyeloma Treatment

The median values of bilirubin, albumin, AST, ALT, GGT and ALP at baseline and after 1 and 3 months of treatment are presented in Table 2. Serum albumin showed a statistically significant monotonic increase over time (*p* < 0.001), most likely reflecting recovery of nutritional status and general condition as a consequence of response to antimyeloma therapy. Aspartate aminotransferase (AST) and gamma-glutamyl transferase (GGT) showed a significant monotonic decrease over time (*p* = 0.018 and *p* = 0.022), with significantly lower values at 3 months compared with baseline for AST, and lower values at 3 months compared with both baseline and 1 month for GGT. Alanine aminotransferase (ALT) did not show a monotonic trend (*p* = 0.191), but values at 3 months were significantly lower than at 1 month. Alkaline phosphatase (ALP) did not show a monotonic trend (*p* = 0.155) but had significantly higher values at 1 month compared with baseline and 3 months (an inverted U-shaped pattern with a transient rise in the first month). Bilirubin showed no statistically significant differences or monotonic trend between time points (*p* > 0.05 for both analyses).

Considered together for any liver marker (AST, ALT, GGT or ALP), normal values at baseline were recorded in 77 (67.0%) patients, a grade 1 abnormality according to CTCAE in 30 (26.1%), grade 2 in five (4.3%) and grade 3 in three (2.6%). After 1 month, normal values were recorded in 64 (59.8%), grade 1 in 32 (29.9%), grade 2 in nine (8.4%) and grade 3 in two (1.9%) patients. After 3 months, normal values were recorded in 76 (76.0%), grade 1 in 20 (20.0%), grade 2 in three (3.0%) and grade 3 in one (1.0%) patient. A statistically significant difference in the proportion of patients with an abnormality was found between 3 months and 1 month (*p* = 0.001).

**Figure 3:**
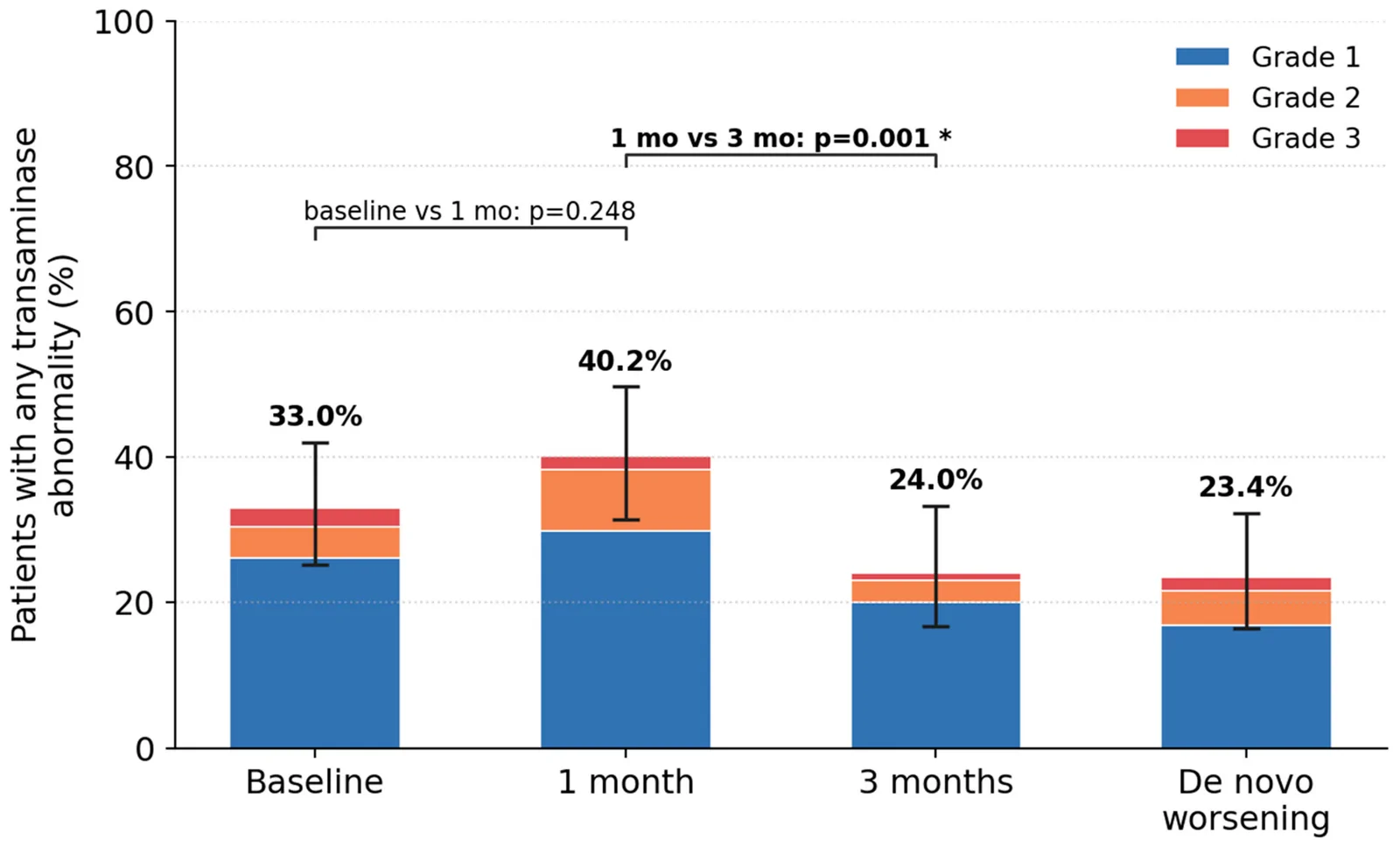
Proportion of patients with normal and elevated transaminases at baseline, at 1 and 3 months after the start of treatment, and with de novo worsening of transaminases. Grades are defined according to CTCAE v5.0. Bars show the proportion of patients with an abnormality of any liver transaminase (AST, ALT, GGT or ALP), stacked by CTCAE v5.0 grade (grade 1, 2 and 3); normal patients (grade 0) make up the remainder to 100%. The “de novo worsening” bar shows patients developing a new or worsening abnormality during treatment. Error bars are 95% confidence intervals for the overall proportion with an abnormality, and *p*-values (McNemar test) are shown for the comparisons between consecutive time points. * depicts statistically significant finding.

De novo worsening of liver enzymes was recorded in 25/107 (23.4%) patients with available laboratory data. In 22/25 (88.0%) patients the peak of worsening occurred at 1 month and in 3/25 (12.0%) at 3 months. De novo worsening was grade 1 in 18/25 (72.0%), grade 2 in 5/25 (20.0%) and grade 3 in 2/25 (8.0%) of affected patients. The pattern of liver injury assessed using the R-ratio indicated a cholestatic pattern in 24/25 (96.0%) patients with worsening and a mixed pattern in 1/25 (4.0%). The median R-ratio at the peak of worsening was 0.36 (IQR 0.27–0.73), consistent with a predominantly cholestatic pattern of injury. De novo appearance of a grade 2 or higher abnormality was recorded in 7/115 (6.1%) patients, while a grade 2 or higher abnormality arising de novo or by worsening of a milder abnormality was recorded in 15/115 (13.0%) patients. The dynamics of these changes are shown in Figure 3.

The dynamics of individual liver function markers were as follows: serum bilirubin was normal at baseline in 109 (95.6%) patients, with a grade 1 abnormality in 5 (4.4%); the proportions remained essentially unchanged at 1 month (102 [95.3%] normal; 5 [4.7%] grade 1; *p* = 0.625 vs. baseline) and at 3 months (94 [95.9%] normal; 4 [4.1%] grade 1; *p* = 1.0 vs. baseline). Serum albumin was normal at baseline in 24 (21.2%), with grade 1 abnormality in 61 (54.0%), grade 2 in 22 (19.5%) and grade 3 in 6 (5.3%); after 3 months the proportion with normal values increased to 49 (49.0%) (*p* < 0.001 vs. baseline; *p* = 0.002 vs. 1 month), consistent with progressive recovery. Serum AST was normal at baseline in 100 (87.0%) and in 97 (97.0%) at 3 months (*p* = 0.008 vs. baseline). Serum ALT was normal at baseline in 102 (88.7%) and in 96 (96.0%) at 3 months (*p* = 0.057 vs. baseline). Serum GGT was normal at baseline in 87 (75.7%), with grade 1 derangements in 21 (18.3%), grade 2 in four (3.5%) and grade 3 in three patients (2.6%); at 3 months 83 (83.0%) were normal (*p* = 0.003 vs. 1 month). Serum ALP was normal at baseline in 107 (93.9%) but only 82 (76.6%) at 1 month (*p* < 0.001 vs. baseline), recovering to 90 (90.0%) at 3 months (*p* = 0.004 vs. 1 month), confirming the transient first-month rise.

### 2.3. Relationship of Antimyeloma and Supportive Therapies with Liver Function Abnormalities

**Figure 4:**
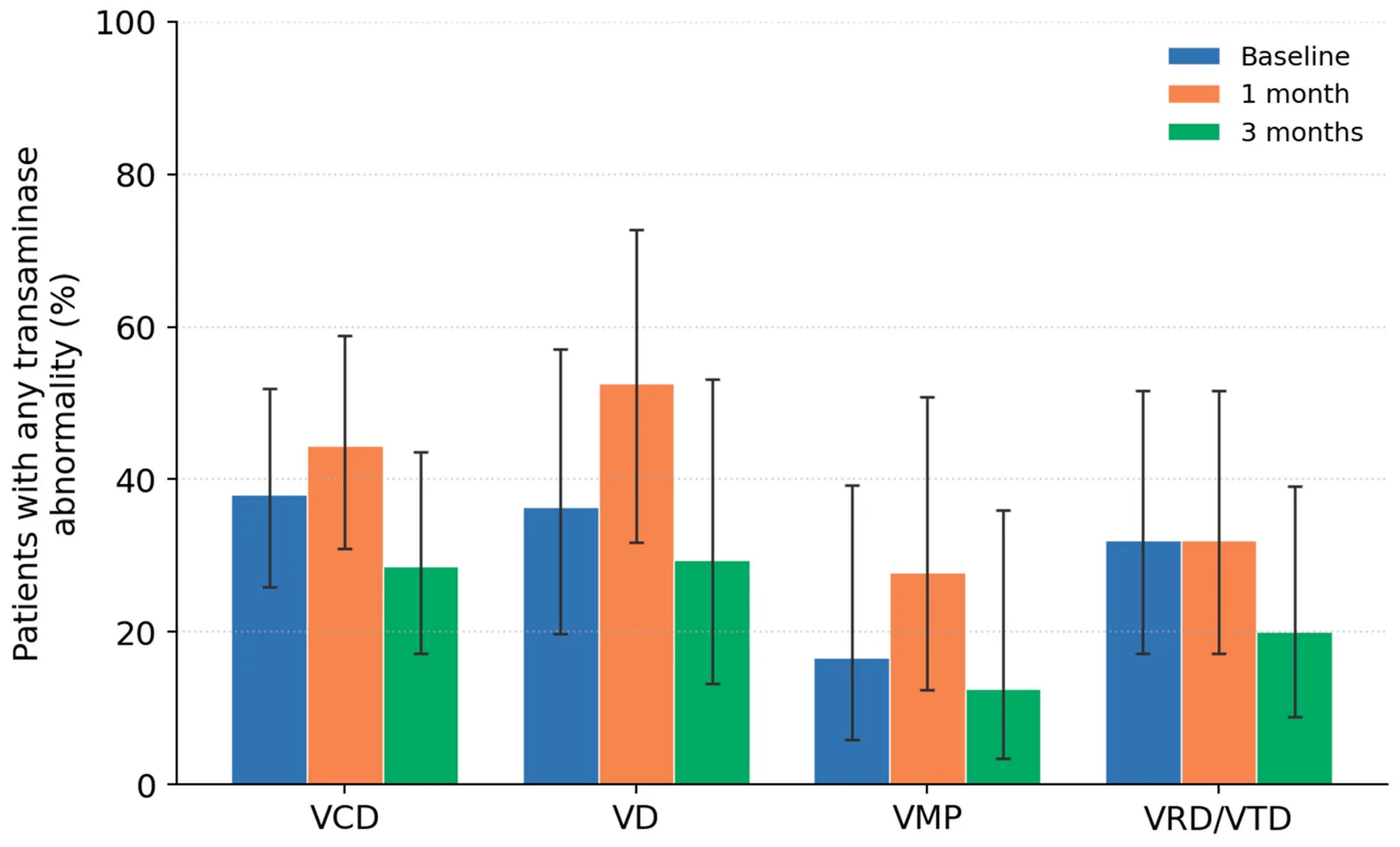
Proportion of patients with an abnormality of any liver transaminase at baseline and 1 and 3 months after the start of treatment, stratified by the antimyeloma regimen administered. VCD: bortezomib, cyclophosphamide, dexamethasone; VD: bortezomib, dexamethasone; VMP: bortezomib, melphalan, prednisone; VRD: bortezomib, lenalidomide, dexamethasone; VTD: bortezomib, thalidomide, dexamethasone. The VRD and VTD groups are shown combined as IMiD-based regimens, as the VTD group comprised only 2 patients.

The relationship between exposure to individual antimyeloma regimens and other supportive therapies and the occurrence of liver injury is presented in Table 3. No statistically significant difference was found in the frequency of liver injury at baseline, 1 month or 3 months, nor in the rate of transaminase worsening during treatment between patients treated with the different antimyeloma regimens (*p* > 0.05 for all analyses). The supportive therapy profile, as well as the predefined hepatotoxicity risk categories of supportive drugs, were likewise not significantly associated with the occurrence of liver injury over the study period (*p* > 0.05 for all analyses). The proportions of patients with transaminase abnormalities at baseline and 1 and 3 months stratified by antimyeloma regimen are shown in Figure 4. When the analysis was repeated at the level of the individual agents contained in the regimens, none of the antimyeloma drugs was significantly associated with a transaminase abnormality at any time point or with de novo worsening (*p* > 0.05 for all analyses). Bortezomib was administered to all patients and therefore could not be evaluated comparatively.

### 2.4. Relationship of Comorbidities and Other Parameters with Liver Function Abnormalities

Analyses of the relationship between comorbidities and other clinical parameters and the occurrence of transaminase abnormalities are presented in Supplementary Tables S3 and S4. Among the analyzed clinical and laboratory characteristics, liver injury at baseline was significantly associated with age ≥ 68 years (odds ratio [OR] 0.44, 95% confidence interval [CI] 0.20–0.97, *p* = 0.043), beta-2-microglobulin ≥ 5.5 mg/L (OR 2.55, 95% CI 1.15–5.65, *p* = 0.021) and ISS stage 3 vs. ISS 1 (OR 7.39, 95% CI 1.55–35.24, *p* = 0.012). Liver injury at 1 month was significantly associated with creatinine ≥ 105 umol/L (OR 2.47, 95% CI 1.11–5.46, *p* = 0.026), beta-2-microglobulin ≥ 5.5 mg/L (OR 3.37, 95% CI 1.50–7.55, *p* = 0.003) and ISS stage 3 vs. ISS 1 (OR 4.14, 95% CI 1.19–14.41, *p* = 0.026).

Liver injury at 3 months was significantly associated with creatinine ≥ 105 umol/L (OR 3.34, 95% CI 1.24–9.00, *p* = 0.017), beta-2-microglobulin ≥ 5.5 mg/L (OR 3.63, 95% CI 1.38–9.58, *p* = 0.009) and light-chain myeloma vs. IgG (OR 3.30, 95% CI 1.01–10.78, *p* = 0.040). De novo worsening of liver function was significantly associated with the presence of a second malignancy (OR 3.55, 95% CI 1.14–11.07, *p* = 0.029), albumin ≥ 36 g/L (OR 2.72, 95% CI 1.05–7.00, *p* = 0.039) and light-chain myeloma vs. IgG (OR 4.61, 95% CI 1.39–15.35, *p* = 0.013).

### 2.5. Independent Predictors of Transaminase Abnormality and De Novo Worsening

Four multivariate logistic regression models with backward elimination were constructed. Each model obligatorily included age, sex, type of antimyeloma regimen (reference = VCD; VTD merged with VRD as an IMiD-based regimen because of the small sample), presence of osteolytic lesions and high hepatotoxicity risk of supportive therapy, with the addition of variables that reached *p* < 0.10 in univariate analysis for the same outcome (Table 3 and Supplementary Tables S3 and S4). In the models for abnormality at 1 and 3 months and for de novo worsening, an abnormality of any transaminase at baseline was additionally included as an obligatory predictor.

In multivariate analysis, the only independently significant predictor of a transaminase abnormality at baseline was ISS stage 3 vs. ISS 1 (OR 4.51, 95% CI 1.55–13.11, *p* = 0.006). The independently significant predictors of an abnormality at 1 month were an abnormality of any transaminase at baseline (OR 8.08, 95% CI 2.92–22.35, *p* < 0.001) and beta-2-microglobulin ≥ 5.5 mg/L (OR 3.61, 95% CI 1.30–10.03, *p* = 0.014). The independently significant predictors of an abnormality at 3 months were an abnormality of any transaminase at baseline (OR 54.15, 95% CI 6.01–488.08, *p* < 0.001), beta-2-microglobulin ≥ 5.5 mg/L (OR 11.54, 95% CI 1.72–77.45, *p* = 0.012), light-chain myeloma vs. IgG (OR 13.55, 95% CI 1.58–116.49, *p* = 0.018), lambda vs. kappa light chain (OR 6.98, 95% CI 1.25–39.00, *p* = 0.027) and a second malignancy (OR 18.04, 95% CI 1.74–186.64, *p* = 0.015). The independently significant predictors of de novo worsening were light-chain myeloma vs. IgG (OR 17.84, 95% CI 3.11–102.26, *p* = 0.001) and age ≥ 68 years (OR 5.68, 95% CI 1.29–24.95, *p* = 0.022).

### 2.6. Clinical Significance of Liver Injury for Treatment Response and Survival

Liver injury at individual time points and its worsening over time were not statistically significantly associated with the probability of achieving a response to first-line treatment (*p* > 0.05 for all analyses; Table 4). However, liver injury present at 3 months after the start of treatment was significantly associated with worse survival (hazard ratio [HR] 3.02, 95% CI 1.50–6.01, *p* = 0.002). This effect persisted in a multivariate Cox regression model adjusted for age, sex and ISS stage, in which liver injury at 3 months (HR 2.83, 95% CI 1.27–6.31, *p* = 0.011) and ISS stage 3 vs. 1 (HR 1.05, 95% CI 1.01–1.09, *p* = 0.025) were mutually independent predictors of worse survival. Among the 25 patients with de novo liver injury, treatment was delayed in 16 (64.0%) and the dose was reduced in five (20.0%), whereas treatment was permanently discontinued in one patient owing to multiple concurrent complications. The liver lesion itself was the direct reason for the delay in only two patients, the remaining delays and dose reductions being attributable to hematologic toxicities, infective complications, polyneuropathy, diarrhea, bleeding or other causes. During the evaluation period, 6 (24.0%) patients were hospitalized, but only one directly because of the liver injury, the others being admitted for deterioration of general condition, infective complications, impaired renal function or dehydration. No deaths attributable to liver failure occurred during follow-up. Overall, adjustments to treatment dosing and timing were driven primarily by hematological and infective toxicities rather than by the hepatic abnormalities.

## 3. Discussion

To our knowledge, this is the first study to analyze the incidence and dynamics of liver function impairment in a real-life cohort of patients with multiple myeloma treated in the first line with bortezomib-based regimens, together with its clinical significance for treatment response and survival. We did not confirm our hypothesis that the occurrence of hepatotoxicity in MM patients differs according to the type of administered therapy. Nevertheless, several important observations emerge from our study.

Our cohort reflects patients from real clinical practice with an indication to start treatment. The median age was 68 years, the proportion of patients with features of advanced disease was above average (ISS stage 3 in 53.7%), the rate of ASCT was relatively low (34.2%), and several different bortezomib-based induction regimens were used, reflecting the need to adapt therapy to the clinical characteristics of individual patients and of the disease itself. The burden of comorbidities (metabolic, cardiovascular and other malignancies) was high and patients were exposed to polypharmacy, which increases the possibility of drug interactions and complicates the selection of optimal therapy. A substantial proportion of these patients would therefore have been ineligible for clinical trials owing to multiple exclusion criteria. The median overall survival of 43 months should be interpreted in the context of patient profile and therapeutic era. The inclusion period (2016–2023) entirely precedes the introduction of daratumumab into first-line treatment in Croatia, which became available through the national health insurance fund for transplant-ineligible patients only in 2024; throughout the observed period, bortezomib-based therapy represented the best available first-line standard. Our median survival is consistent with large European registries enrolling a similar population, such as the Czech Myeloma Group Registry of Monoclonal Gammopathies (median OS 46.2 from the initiation of first therapy) [21], the English Cancer Analysis System registry (median OS 44.5 months at first line) [22], a Finnish real-world registry of non-transplant patients (median OS 41.3–43.8 months over 2013–2022) [23], and the EMEA TriNetX cohort (median OS approximately 35–54 months depending on era) [24], while our population had a considerably higher proportion of patients with high ISS stage. Our data therefore cannot be directly compared with published outcomes of the daratumumab-based induction regimens that are becoming the current standard. We deliberately selected this population to reduce the heterogeneity of specific therapies and to attempt to disentangle the contribution of bortezomib and other co-administered drugs to liver injury.

The potential hepatotoxicity of bortezomib is recognized in the literature. The proportion of patients with an abnormality of any liver transaminase in our cohort was 33% at baseline, 40.2% at 1 month and 24% at 3 months. Although transaminase elevations are listed within the broader safety profile of the pivotal randomized bortezomib trials, their precise incidence has not been consistently reported in the primary publications, limiting direct numerical comparison [6,7,8]. The predominantly cholestatic pattern of liver injury that we observed in patients with de novo worsening is consistent with the proposed mechanism of action of bortezomib on hepatocytes and the biliary system. Proteasome inhibition is canonically understood to block the degradation of IκBα and the consequent activation of NF-κB, a transcription factor that in hepatocytes induces the expression of cytoprotective genes [25]. Preclinical toxicology data, primarily from regulatory submissions, have reported hepatocellular hypertrophy, vacuolization and pigment deposition in the liver following bortezomib exposure, while published peer-reviewed preclinical work has more extensively characterized effects on hepatic stellate cell apoptosis and bile duct injury models [9]. Clinical case reports typically describe a rise in transaminases on the third day after the second dose of the first cycle with transient recovery after discontinuation [9,10]. Hepatic toxicity, particularly veno-occlusive disease/sinusoidal obstruction syndrome (VOD/SOS), is a well-recognized complication of the transplant setting, primarily attributed to myeloablative conditioning regimens [26]. Whether proteasome inhibitors independently contribute to hepatic injury in this context remains unclear. Beyond disrupted NF-κB signaling, additional mechanisms proposed to contribute to proteasome inhibitor-associated hepatic injury include accumulation of misfolded proteins with endoplasmic reticulum stress and activation of the unfolded protein response, mitochondrial dysfunction, generation of reactive oxygen species with oxidative stress, and pro-inflammatory cytokine signaling [27,28,29,30].

In our study, a total of 23.4% of patients developed de novo liver injury. Most patients with de novo worsening developed it within the first month of treatment, with only a minority doing so later. These patients showed a predominantly cholestatic pattern, suggestive of biliary injury. Transaminase abnormalities were not significantly associated with the probability of response to therapy, but elevated transaminases at 3 months after the start of treatment were significantly associated with worse survival, independently of the ISS stage, which argues for the importance of detecting and actively addressing such abnormalities when they persist. An evident or occult secondary malignancy should also be recognized as a potential independent contributor to persistent transaminase derangement at 3 months in this older population and may in part explain the association between the 3-month hepatic endpoint and reduced overall survival. Its presence should therefore be actively considered in the diagnostic work-up of patients with sustained liver function abnormalities. The absence of an association with response rates also suggests that the appearance of liver injury should not be used as a marker of response to bortezomib. Reassuringly, the hepatic abnormalities we observed were predominantly mild, and in this cohort they did not in themselves drive modifications of treatment dosing or schedule, which were instead dictated mainly by hematological and infective toxicities. All analyzed regimens also contained a corticosteroid (dexamethasone or prednisone), which can promote hepatic steatosis and mild transaminase elevations, so the abnormalities we observed reflect the combined regimen rather than bortezomib in isolation. Furthermore, because the attribution of liver injury predominantly to the underlying disease rests on the absence of an association with specific regimens or supportive drugs together with indirect correlations with tumor burden markers, rather than on direct histological confirmation, alternative or contributory mechanisms—including concomitant hepatotoxic comedication, metabolic-dysfunction-associated steatotic liver disease, ischemic or sepsis-associated hepatic injury, and unrecognized viral or autoimmune liver disease—cannot be excluded and warrant evaluation in future studies.

A mechanistic explanation for the presence or development of new liver injury remains unresolved. Multiple causes are possible; however, toxicity of antimyeloma therapy is excluded as a possible cause of liver injury already present at baseline. Baseline liver injury, in turn, proved to be the main and very strong predictor of injury present at 1 and 3 months of therapy. The profile of independent predictors of liver injury identified in our study at the various time points, which reflect either increased tumor burden or are otherwise directly related to the biology of multiple myeloma (beta-2-microglobulin, ISS stage 3, light-chain phenotype), points to an important role of the myeloma itself in the development of liver injury. Infiltration of the hepatic parenchyma by plasma cells has been described in 25–40% of autopsy series of MM patients, most often without jaundice or clinically overt symptoms but with a laboratory signal in the form of rising GGT and ALP and hypoalbuminemia [31,32]. High beta-2-microglobulin and ISS stage 3 correlate with the extent of potential extramedullary involvement [33].

Light-chain myeloma was identified as a potential risk factor for the occurrence or persistence of liver injury at later time points in our study. Light-chain deposition, whether in the form of systemic AL amyloidosis (present in approximately 10–15% of MM patients, of whom a subset develops hepatic involvement manifesting with a typical isolated rise in alkaline phosphatase) or light-chain deposition disease, manifests with a cholestatic pattern [34,35], and hepatic plasma cell infiltration with light-chain restriction has been documented as a cause of cholestatic liver enzyme abnormalities [36].

Renal dysfunction may also promote the development of liver injury through several mechanisms. Cast nephropathy, AL amyloidosis and hypercalcemia lead to acute kidney injury, which may secondarily promote systemic inflammation with cytokine-mediated injury, oxidative stress, uremic toxicity, impaired biliary transport and altered drug metabolism [37]. In our study, creatinine showed an association with liver injury at baseline and at 1 month, but most likely owing to its correlation with beta-2-microglobulin it lost statistical significance after adjustment in the multivariate models.

It is also important to emphasize that background comorbidities and polypharmacy may be associated with the development of liver injury, even though we were unable to demonstrate statistical significance in our study. The high prevalence of non-alcoholic fatty liver disease in the age group above 65 years, polypharmacy with LiverTox category A-B drugs (statins, co-trimoxazole, azole antifungals, allopurinol) and undiagnosed chronic comorbidities could additionally explain the significant laboratory abnormalities and worse prognosis in real-life cohorts [18,19], and prophylactic and therapeutic antimicrobial exposure is particularly frequent in this immunocompromised population [38].

The main limitations of our work are its retrospective design, which precludes causal inference, and the fact that our results are representative of the experience of a single tertiary center evaluating consecutive patients with multiple comorbidities and advanced clinical characteristics. Follow-up was limited to three time points in the early phase of treatment, the study was based on laboratory evaluation, and imaging and histopathological assessment of the liver were not available within the scope of this research. In addition, formal causality assessment (e.g., the RUCAM score) was neither performed nor reconstructable retrospectively. Pre-existing liver disease beyond the excluded active hepatitis B or C and known chronic liver disease, alcohol consumption, and the work-up of abnormal findings (imaging, steatotic and autoimmune disease, hepatitis E or B reactivation) were assessed at the physician’s discretion rather than systematically, so unrecognized concurrent liver conditions cannot be excluded. Although liver-related treatment modifications and hospitalizations were recorded and were only infrequently attributable to the liver injury itself, the predominantly mild, laboratory-defined nature of the abnormalities and the absence of formal causality assessment still preclude a firm distinction between isolated laboratory changes and clinically significant liver injury. The cohort reflects Croatian practice from 2016 to 2023, before routine first-line daratumumab, limiting generalizability to daratumumab-based regimens. Finally, some multivariate estimates had wide confidence intervals, reflecting the limited sample size and event numbers, and indicate the direction rather than the precise magnitude of association.

Nevertheless, this is the first study to systematically analyze liver function impairment measured by laboratory findings in MM patients treated with bortezomib-based regimens, and the unique results we describe need to be confirmed in prospective and independent patient cohorts.

## 4. Materials and Methods

### 4.1. Patients and Study Design

We retrospectively analyzed patients with multiple myeloma consecutively treated at University Hospital Dubrava in Zagreb between 2016 and 2023 who received first-line bortezomib-based regimens. Patients with a history of chronic liver disease, active hepatitis B or C virus infection, or an insufficient number of available laboratory findings were excluded. The study was conducted in accordance with the principles of the Declaration of Helsinki and approved by the institutional ethics committee.

Patients were treated with the following regimens: VCD (bortezomib, cyclophosphamide and dexamethasone), VMP (bortezomib, melphalan and prednisone), VD (bortezomib and dexamethasone), VRD (bortezomib, lenalidomide and dexamethasone) and VTD (bortezomib, thalidomide and dexamethasone). A proportion of patients were treated with ASCT. Response to first-line treatment was assessed according to the consensus criteria of the International Myeloma Working Group (IMWG) [39,40]. Response categories were complete response (CR), very good partial response (VGPR), partial response (PR), stable disease (SD) and progressive disease (PD). Overall survival was recorded from the date of treatment initiation to the last follow-up or death.

At the start of treatment, standard demographic, clinical and laboratory parameters related to multiple myeloma were recorded (sex, age, ISS stage, heavy-chain type, light-chain type, presence of osteolytic lesions, hemoglobin, leukocytes, platelets, creatinine, calcium, albumin, beta-2-microglobulin, lactate dehydrogenase, free light-chain kappa/lambda ratio, percentage of bone marrow plasma cells), as well as patient comorbidities. Liver function laboratory parameters (serum bilirubin, albumin, aspartate aminotransferase [AST], alanine aminotransferase [ALT], gamma-glutamyl transferase [GGT] and alkaline phosphatase [ALP]) were recorded at baseline and after 1 and 3 months from the start of treatment.

### 4.2. Liver Function Assessment and Grading

Grades of liver function abnormality were defined according to the CTCAE version 5.0 criteria [20]. Grade 0 denotes values within the reference range, and grades 1–4 are as follows. Bilirubin (rise above the upper limit of normal, ULN): grade 1, up to 1.5× ULN; grade 2, 1.5–3× ULN; grade 3, 3–10× ULN; grade 4, more than 10× ULN. Albumin (fall below the lower limit of normal): grade 1, below normal to 30 g/L; grade 2, 30–20 g/L; grade 3, below 20 g/L; grade 4, life-threatening outcomes. AST and ALT (rise above ULN): grade 1, up to 3× ULN; grade 2, 3–5× ULN; grade 3, 5–20× ULN; grade 4, more than 20× ULN. GGT and ALP (rise above ULN): grade 1, up to 2.5× ULN; grade 2, 2.5–5× ULN; grade 3, 5–20× ULN; grade 4, more than 20× ULN.

As primary outcomes of interest we selected an abnormality of any liver transaminase (AST, ALT, GGT or ALP) of grade ≥ 1, assessed at each time point (baseline, 1 month and 3 months), and worsening of grade or appearance of a new abnormality at later time points. The pattern of liver injury in patients with worsening was assessed using the R-ratio [R = (ALT/ULN)/(ALP/ULN)] according to the consensus definition of the DILI Expert Working Group [41,42]: R ≥ 5 denotes a hepatocellular pattern, R ≤ 2 a cholestatic pattern, and 2 < R < 5 a mixed pattern of injury [42]. Drugs used in supportive therapy were classified according to hepatotoxicity risk using the LiverTox database [19], in which drugs are categorized into classes A-E according to the number of published cases of hepatotoxicity; for the purposes of analysis they were grouped into high-risk (LiverTox categories A and B), moderate-risk (LiverTox C) and low-risk (LiverTox D and E) hepatotoxicity.

### 4.3. Statistical Analysis

The normality of distribution of numerical variables was analyzed with the Shapiro–Wilk test. Categorical variables were described by absolute numbers and percentages, and continuous variables by median with interquartile range (IQR). Survival analysis was based on the Kaplan–Meier method. Differences in individual liver function parameters between measurement time points were analyzed with the Friedman ANOVA test with the Wilcoxon post hoc test, while the trend of increase or decrease was analyzed with the Page L test. Differences in the proportion of patients with an abnormality between time points were analyzed with the McNemar test. The association of the studied variables with the occurrence of liver toxicity was analyzed by univariate and multivariate logistic regression separately for each of the four outcomes (abnormality of any transaminase at baseline, at 1 month and at 3 months, and de novo worsening). Each model included the type of therapeutic regimen, age, sex, the hepatotoxicity grade of supportive therapy and the presence of osteolytic lesions, with the addition of variables that reached *p* < 0.10 in univariate analysis. The association of liver injury categories with survival was performed using Cox regression. *p*-values < 0.05 were considered statistically significant. Analyses were performed using MedCalc Statistical Software version 23.5.2 (MedCalc Software Ltd., Ostend, Belgium) and the Python 3 programming language (Python Software Foundation, Wilmington, DE, USA) with the pandas, NumPy, SciPy, statsmodels and lifelines libraries.

## 5. Conclusions

Liver function abnormalities are frequent in real-life patients with multiple myeloma and are already present before antimyeloma therapy in 33% of patients, indicating that much of the liver signal reflects the underlying disease rather than the treatment itself. During the first three months of bortezomib-based treatment liver transaminases follow a gradually regressive course, accompanied by one-month transitory ALP increase, while de novo liver injury develops in 23.4% of patients, most often within the first month and with a predominantly cholestatic pattern. A baseline transaminase abnormality is the strongest predictor of later abnormalities, whereas the antimyeloma regimen, the hepatotoxicity risk of supportive therapy and most comorbidities show no independent association (except for second malignancy for the 3-month timepoint). Liver abnormalities are not associated with treatment response. Importantly, an abnormality persisting at 3 months independently predicts worse overall survival, supporting active monitoring of liver function during bortezomib-based treatment.

## Figures and Tables

**Table 1:** Clinical and laboratory characteristics of patients with multiple myeloma.

Characteristic	Value
Male sex	63/115 (54.8%)
Age (years)	68 (60–74)
ISS stage 1	20/95 (21.1%)
ISS stage 2	24/95 (25.3%)
ISS stage 3	51/95 (53.7%)
Heavy-chain–non-secretory	3/113 (2.7%)
Heavy chain–IgG	74/113 (65.5%)
Heavy chain–IgA	19/113 (16.8%)
Heavy chain–light-chain only	17/113 (15.0%)
Light-chain–kappa	73/111 (65.8%)
Light-chain–lambda	37/111 (33.3%)
First-line regimen–VCD	50/115 (43.5%)
First-line regimen–VD	22/115 (19.1%)
First-line regimen–VMP	18/115 (15.7%)
First-line regimen–VRD	23/115 (20.0%)
First-line regimen–VTD	2/115 (1.7%)
ASCT–none	73/111 (65.8%)
ASCT–single	26/111 (23.4%)
ASCT–tandem	12/111 (10.8%)
Hemoglobin (g/L)	106 (91–121)
Osteolytic lesions	86/111 (77.5%)
Creatinine (umol/L)	105 (71.5–213.5)
Calcium (mmol/L)	2.4 (2.22–2.54)
Platelets (×10^9^/L)	222 (184–275.5)
Leukocytes (×10^9^/L)	6.9 (5.35–8.95)
Albumin (g/L)	36 (31–39.75)
Beta-2-microglobulin (mg/L)	5.5 (3.2–9.83)
Free light-chain ratio kappa/lambda	4 (0.08–26.97)
Bone marrow plasma cells (%)	48 (23–72.75)
LDH (U/L)	176 (146–214)

Numerical variables are presented as median and interquartile range in parentheses. ISS: International Staging System; IgG: immunoglobulin G; IgA: immunoglobulin A; VCD: bortezomib, cyclophosphamide, dexamethasone; VD: bortezomib, dexamethasone; VMP: bortezomib, melphalan, prednisone; VRD: bortezomib, lenalidomide, dexamethasone; VTD: bortezomib, thalidomide, dexamethasone; ASCT: autologous stem cell transplantation; CR: complete response; VGPR: very good partial response; PR: partial response; SD: stable disease; PD: progressive disease; LDH: lactate dehydrogenase.

**Table 2:** Liver function parameters recorded at baseline and after 1 and 3 months of treatment.

Parameter	Baseline	1 Month	3 Months	*p* (Difference)	*p* (Trend)
Bilirubin (umol/L)	9.4 (6.7–11.2)	9.1 (6.8–12.1)	9.3 (7.1–11.7)	0.569	0.720
Albumin (g/L)	35 (30–39)	36 (33–40)	39 (36–42)	<0.001 *^,†^	<0.001 (rise)
AST (U/L)	21 (17–29)	20 (16–26.5)	20 (16–24)	0.046 *	0.018 (fall)
ALT (U/L)	19.5 (13–32.5)	21 (15–32)	18 (13–24)	0.037 ^†^	0.191
GGT (U/L)	29.5 (17–52)	28.5 (19–58)	24 (17–39)	0.001 *^,†^	0.022 (fall)
ALP (U/L)	76 (57–91.8)	96 (78–136.3)	80 (59.5–101.5)	<0.001 ^†,‡^	0.155

Values are presented as median and interquartile range. Pairwise comparisons (Wilcoxon post hoc): * 3 months vs. baseline; ^†^ 3 months vs. 1 month; ^‡^ 1 month vs. baseline. AST: aspartate aminotransferase; ALT: alanine aminotransferase; GGT: gamma-glutamyl transferase; ALP: alkaline phosphatase.

**Table 3:** Proportion of patients with an abnormality of any liver transaminase (AST, ALT, GGT or ALP) at baseline and 1 and 3 months, and with de novo worsening during treatment, stratified by antimyeloma regimen, supportive drug hepatotoxicity risk class and individual supportive drug categories.

Subgroup	Baseline	1 Month	3 Months	New Worsening
VCD	19/50 (38.0%)	20/45 (44.4%)	12/42 (28.6%)	11/45 (24.4%)
VD	8/22 (36.4%)	10/19 (52.6%)	5/17 (29.4%)	5/19 (26.3%)
VMP	3/18 (16.7%)	5/18 (27.8%)	2/16 (12.5%)	3/18 (16.7%)
VRD	8/23 (34.8%)	8/23 (34.8%)	5/23 (21.7%)	6/23 (26.1%)
VTD	0/2 (0%)	0/2 (0%)	0/2 (0%)	0/2 (0%)
(*p*-value, regimen)	*p* = 0.315	*p* = 0.490	*p* = 0.649	*p* = 0.779
IMiD-based	8/25 (32.0%)	8/25 (32.0%)	5/25 (20.0%)	6/25 (24.0%)
Non-IMiD	30/90 (33.3%)	35/82 (42.7%)	19/75 (25.3%)	19/82 (23.2%)
(*p*-value, IMiD)	*p* = 0.900	*p* = 0.530	*p* = 0.904	*p* = 0.932
High hepatotoxicity risk (support. therapy)	27/78 (34.6%)	29/72 (40.5%)	19/68 (27.9%)	18/74 (24.3%)
Moderate hepatotoxicity risk (support. therapy)	10/31 (32.3%)	11/29 (37.9%)	5/28 (17.9%)	6/29 (20.7%)
Low hepatotoxicity risk (support. therapy)	1/4 (25.0%)	2/4 (50.0%)	0/4 (0%)	1/4 (25.0%)
(*p*-value, risk class)	*p* = 0.905	*p* = 0.849	*p* = 0.185	*p* = 0.937
Supportive drug category, users (*p* vs. non-users)				
Bisphosphonates	28/77 (36.4%); *p* = 0.283	32/77 (41.6%); *p* = 0.191	17/77 (22.1%); *p* = 0.650	17/74 (23.0%); *p* = 0.886
Cardiovascular drugs	22/73 (30.1%); *p* = 0.383	25/73 (34.2%); *p* = 0.359	14/73 (19.2%); *p* = 0.557	17/69 (24.6%); *p* = 0.675
Gastroprotective drugs	31/88 (35.2%); *p* = 0.371	35/88 (39.8%); *p* = 0.343	20/88 (22.7%); *p* = 0.380	19/82 (23.2%); *p* = 0.932
Analgesics/opioids	21/60 (35.0%); *p* = 0.641	25/60 (41.7%); *p* = 0.323	9/60 (15.0%); *p* = 0.110	12/58 (20.7%); *p* = 0.478
Antimicrobial drugs	28/81 (34.6%); *p* = 0.592	31/81 (38.3%); *p* = 0.763	16/81 (19.8%); *p* = 0.650	14/75 (18.7%); *p* = 0.083
Antidiabetic/endocrine drugs	9/31 (29.0%); *p* = 0.579	11/31 (35.5%); *p* = 0.797	5/31 (16.1%); *p* = 0.449	8/30 (26.7%); *p* = 0.615
Neurological/psychiatric drugs	13/37 (35.1%); *p* = 0.743	13/37 (35.1%); *p* = 0.731	7/37 (18.9%); *p* = 0.723	6/35 (17.1%); *p* = 0.292
Anticoagulant/antiplatelet drugs	12/39 (30.8%); *p* = 0.710	13/39 (33.3%); *p* = 0.520	9/39 (23.1%); *p* = 0.677	8/36 (22.2%); *p* = 0.842
Other therapy	31/84 (36.9%); *p* = 0.152	33/84 (39.3%); *p* = 0.490	20/84 (23.8%); *p* = 0.209	18/78 (23.1%); *p* = 0.908

Data are n/N (%). For antimyeloma regimens, the immunomodulatory drug grouping and the supportive drug hepatotoxicity risk class, the *p*-value compares the subgroups within each grouping; for individual supportive drug categories, each proportion is followed by the *p*-value for the comparison between patients exposed vs. not exposed to that category. VCD: bortezomib, cyclophosphamide, dexamethasone; VD: bortezomib, dexamethasone; VMP: bortezomib, melphalan, prednisone; VRD: bortezomib, lenalidomide, dexamethasone; VTD: bortezomib, thalidomide, dexamethasone; IMiD: immunomodulatory drug; AST: aspartate aminotransferase; ALT: alanine aminotransferase; GGT: gamma-glutamyl transferase; ALP: alkaline phosphatase.

**Table 4:** Relationship of liver function abnormalities with response rates and overall survival.

Liver Injury Time Point	Response (CR/VGPR/PR)	Overall Survival
Baseline	*p* = 0.715	*p* = 0.098
1 month	*p* = 0.179	*p* = 0.271
3 months	*p* = 0.748	HR 3.02 (1.50–6.01), *p* = 0.002
De novo worsening	*p* = 0.624	*p* = 0.433

Response analyzed by logistic regression; overall survival using Cox regression. HR: hazard ratio; CR: complete response; VGPR: very good partial response; PR: partial response.

## Data Availability

The data presented in this study are available on reasonable request from the corresponding author. The data are not publicly available owing to privacy and ethical restrictions.

## Supplementary Materials

The following supporting information can be downloaded at: https://www.mdpi.com/article/10.3390/ph19081205/s1. Table S1: Comorbidities of patients with multiple myeloma; Table S2: Profile of supportive therapy administered during the first three months of treatment; Table S3: Proportion of patients with an abnormality of any liver transaminase at baseline and 1 and 3 months and with de novo worsening, stratified by comorbidities; Table S4: Proportion of patients with an abnormality of any liver transaminase at baseline and 1 and 3 months and with de novo worsening, stratified by clinical and laboratory characteristics.
