# Supplementary material for: Hepatotoxicity Profile of Bortezomib-Containing Regimens for First-Line Multiple Myeloma Treatment
Source: Pharmaceuticals (Basel). 2026 Aug 1;19(8):1205. doi: 10.3390/ph19081205 (PMC13516933; doi:10.3390/ph19081205)
Supplement: Supplementary file 1 [file pharmaceuticals-19-01205-s001.zip › pharmaceuticals-4382278-supplementary.pdf]

Supplementary Materials

Table S1. Comorbidities of patients with multiple myeloma.

Comorbidity	Patients
Atrial fibrillation	5/114 (4.4%)
Heart failure	16/114 (14.0%)
Arterial hypertension	68/114 (59.6%)
Diabetes mellitus	23/114 (20.2%)
COPD	6/113 (5.3%)
Smoking	21/111 (18.9%)
Obesity	9/97 (9.3%)
Second malignancy	15/113 (13.3%)
Acute infection	7/63 (11.1%)

COPD: chronic obstructive pulmonary disease.

Table S2. Profile of supportive therapy administered during the first three months of treatment.

Drug category	Representative individual agents	Patients
Bisphosphonates and other bone-metabolism agents	zoledronate, pamidronate, denosumab, others (ibandronate, risedronate)	77/115 (67.0%)
Cardiovascular drugs	amlodipine, atenolol, atorvastatin, bisoprolol, diltiazem, hydrochlorothiazide, indapamide, isosorbide mononitrate, carvedilol, lisinopril, losartan, nebivolol, nifedipine, perindopril, propranolol, ramipril, rosuvastatin, simvastatin, valsartan, verapamil, and others	73/115 (63.5%)
Gastroprotective drugs	Saccharomyces boulardii, antacids (Al/Mg hydroxide), bisacodyl, esomeprazole, famotidine, lactulose, mesalazine, metoclopramide, pantoprazole, probiotics, ranitidine, sevelamer, and others	88/115 (76.5%)
Analgesics/opioids	buprenorphine, dexketoprofen, diclofenac, fentanyl, ibuprofen, ketoprofen, morphine, paracetamol, pregabalin, tapentadol, tramadol	60/115 (52.2%)
Antimicrobial drugs	acyclovir, amoxicillin, ceftriaxone, cefuroxime, ciprofloxacin, fluconazole, clavulanic acid, meropenem, metronidazole, moxifloxacin, piperacillin/tazobactam, sulfamethoxazole/trimethoprim, vancomycin	81/115 (70.4%)
Antidiabetic/endocrine drugs	empagliflozin, gliclazide, human insulin and insulin analogues, levothyroxine, liraglutide, metformin, sitagliptin	31/115 (27.0%)
Neurological/psychiatric drugs	alprazolam, diazepam, duloxetine, escitalopram, phenytoin, phenobarbital, carbamazepine, clonazepam, quetiapine, lorazepam, mirtazapine, olanzapine, venlafaxine, zolpidem, and others	37/115 (32.2%)
Anticoagulant/antiplatelet drugs	acetylsalicylic acid, apixaban, dabigatran, dalteparin, clopidogrel, nadroparin, rivaroxaban, ticagrelor, warfarin	39/115 (33.9%)
Other therapy	allopurinol, febuxostat, budesonide, cyanocobalamin, desloratadine, enteral nutrition, finasteride, furosemide, montelukast, salbutamol, tamsulosin, B-group vitamins, ferrous fumarate, and others	84/115 (73.0%)

The 'Patients' column shows the number and proportion (n/N (%)) of patients who received at least one agent from the corresponding drug category during the first three months of treatment. Al/Mg: aluminium/magnesium.

Table S3. Proportion of patients with an abnormality of any liver transaminase at baseline and 1 and 3 months, and with de novo worsening, stratified by comorbidities.

Comorbidity	Baseline	1 month	3 months	New worsening
Atrial fibrillation	1/5 (20.0%)	1/5 (20.0%)	1/5 (20.0%)	1/4 (25.0%)
Heart failure	4/16 (25.0%)	8/16 (50.0%)	5/16 (31.2%)	5/15 (33.3%)
Arterial hypertension	23/68 (33.8%)	26/68 (38.2%)	15/68 (22.1%)	16/63 (25.4%)
Diabetes mellitus	7/23 (30.4%)	8/23 (34.8%)	2/23 (8.7%)	5/22 (22.7%)
COPD	3/6 (50.0%)	2/6 (33.3%)	1/6 (16.7%)	0/6 (0%)
Smoking	10/21 (47.6%)	8/21 (38.1%)	5/21 (23.8%)	2/21 (9.5%)
Obesity	5/9 (55.6%)	3/9 (33.3%)	2/9 (22.2%)	3/9 (33.3%)
Second malignancy	4/15 (26.7%)	8/15 (53.3%)	6/15 (40.0%)	7/15 (46.7%) *
Acute infection	3/7 (42.9%)	1/7 (14.3%)	2/7 (28.6%)	0/6 (0%)

Data are n/N (%). * p=0.029. All other comparisons p>0.05. COPD: chronic obstructive pulmonary disease.

Table S4. Proportion of patients with an abnormality of any liver transaminase at baseline and 1 and 3 months, and with de novo worsening, stratified by clinical and laboratory characteristics.

Characteristic	Baseline	1 month	3 months	New worsening
Male sex	23/63 (36.5%)	25/63 (39.7%)	13/63 (20.6%)	11/59 (18.6%)
Age >=68 years	14/58 (24.1%) *	19/58 (32.8%)	10/58 (17.2%)	13/52 (25.0%)
Hemoglobin >=106 g/L	17/58 (29.3%)	18/58 (31.0%)	11/58 (19.0%)	12/54 (22.2%)
Osteolytic lesions	32/86 (37.2%)	35/86 (40.7%)	19/86 (22.1%)	20/81 (24.7%)
Creatinine >=105 umol/L	23/58 (39.7%)	27/58 (46.6%) *	17/58 (29.3%) *	14/53 (26.4%)
Calcium >=2.4 mmol/L	15/46 (32.6%)	20/46 (43.5%)	12/46 (26.1%)	13/41 (31.7%)
Platelets >=222 x10^9/L	19/58 (32.8%)	19/58 (32.8%)	10/58 (17.2%)	11/53 (20.8%)
Leukocytes >=6.9 x10^9/L	22/58 (37.9%)	23/58 (39.7%)	13/58 (22.4%)	12/54 (22.2%)
Albumin >=36 g/L	15/58 (25.9%)	21/58 (36.2%)	12/58 (20.7%)	17/53 (32.1%) *
Beta-2-microglobulin >=5.5 mg/L	22/49 (44.9%) *	26/49 (53.1%) *	16/49 (32.7%) *	14/46 (30.4%)
FLC ratio >=4	14/47 (29.8%)	16/47 (34.0%)	8/47 (17.0%)	12/44 (27.3%)
Bone marrow plasma cells >=48%	21/55 (38.2%)	21/55 (38.2%)	10/55 (18.2%)	8/49 (16.3%)
LDH >=176 U/L	21/58 (36.2%)	23/58 (39.7%)	15/58 (25.9%)	14/52 (26.9%)
ISS 1 (ref.)	2/20 (10.0%)	4/20 (20.0%)	4/20 (20.0%)	4/18 (22.2%)
ISS 2	6/24 (25.0%)	7/24 (29.2%)	3/24 (12.5%)	4/24 (16.7%)
ISS 3	23/51 (45.1%) *	26/51 (51.0%) *	16/51 (31.4%)	14/48 (29.2%)
IgG (ref.)	23/74 (31.1%)	26/74 (35.1%)	13/74 (17.6%)	11/69 (15.9%)
IgA	4/19 (21.1%)	4/19 (21.1%)	2/19 (10.5%)	4/18 (22.2%)
Light-chain myeloma	8/17 (47.1%)	9/17 (52.9%)	7/17 (41.2%) *	7/15 (46.7%) *
Kappa (ref.)	22/73 (30.1%)	24/73 (32.9%)	11/73 (15.1%)	15/68 (22.1%)
Lambda	13/37 (35.1%)	15/37 (40.5%)	11/37 (29.7%)	7/34 (20.6%)

Data are n/N (%); ref.: reference category. * Statistically significant association (p<0.05) in univariate analysis. FLC: free light chains; LDH: lactate dehydrogenase; ISS: International Staging System; IgG: immunoglobulin G; IgA: immunoglobulin A.
